# Coronary artery dissection and acute myocardial infarction following blunt chest trauma

**DOI:** 10.1186/1749-7922-4-14

**Published:** 2009-04-14

**Authors:** Johannes L Bjørnstad, Johan Pillgram-Larsen, Theis Tønnessen

**Affiliations:** 1 Department of Cardiothoracic Surgery, Ullevål University Hospital, NO-0407 Oslo, Norway

## Abstract

Blunt chest trauma might lead to cardiac injury ranging from simple arrhythmias to lethal conditions such as cardiac rupture. We experienced a case of initially overlooked traumatic coronary artery dissection which resulted in acute myocardial infarction (AMI). A high degree of suspicion is needed to diagnose this condition. Based on our case, we will give an overview of relevant literature on this topic. ECG, echocardiography, coronary angiography and cardiac enzymes are valuable tools in diagnosing this rare condition. The time span from coronary artery occlusion to revascularisation must be short if AMI is to be avoided.

## Review

Blunt chest trauma might lead to cardiac injury ranging from simple arrhythmias to lethal conditions such as cardiac rupture. Acute myocardial infarction (AMI) may be induced by blunt chest trauma [1-3]. We experienced a case of coronary artery dissection with subsequent myocardial infarction from blunt chest trauma. We will give an overview of relevant literature regarding this topic.

**Figure 1:**
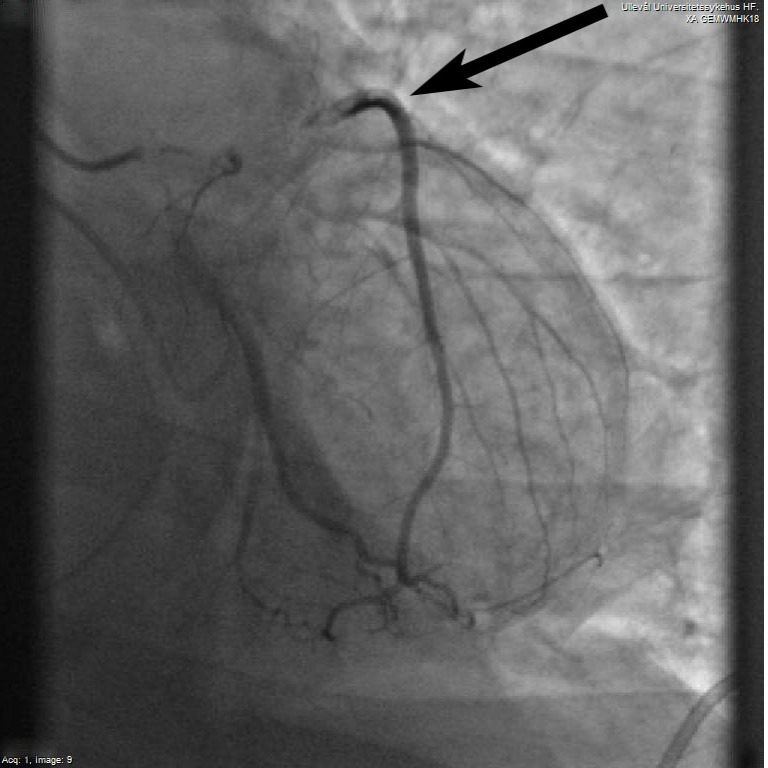
**Coronary angiogram showing dissection of the middle part of the left anterior descending coronary artery (arrow)**.

Parmley reported on 546 autopsy cases of blunt heart injury, and there were nine cases of coronary artery rupture and one case of intimal laceration [4]. None of the cases, however, showed signs of coronary artery occlusion. AMI as a result of coronary artery dissection has been considered rare [3], however coronary artery dissection from blunt trauma has been more frequently described recently [5-15]. This might indicate that this condition previously has been underdiagnosed or is increasing in incidence. The left anterior descending coronary artery (LAD) is the vessel most often affected, and road traffic accidents are the usual cause of traumatic myocardial infarction [3,16]. This susceptibility is attributable to the LAD's anatomic relation to the anterior chest wall allowing both direct trauma and deceleration as possible mechanisms of trauma [16]. In our case the patient suffered blunt chest trauma as his car collided with a moose. He experienced dissection of the middle part of the LAD (Figure 1). Both coronary artery dissection, intimal tear, plaque rupture or epicardial hematoma might lead to AMI after blunt trauma. However, in 12 published cases of traumatic AMI the coronary angiograms were completely normal [3]. Spasm or lysis of a thrombus might explain AMI in these cases. It should be noted that AMI also has been reported after mild trauma [13,17,18].

**Figure 2:**
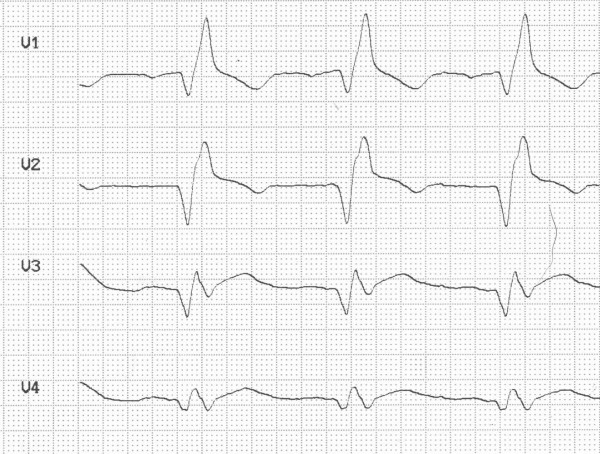
**Electrocardiogram on admission showing sinus rhythm and right bundle branch block**.

**Figure 3:**
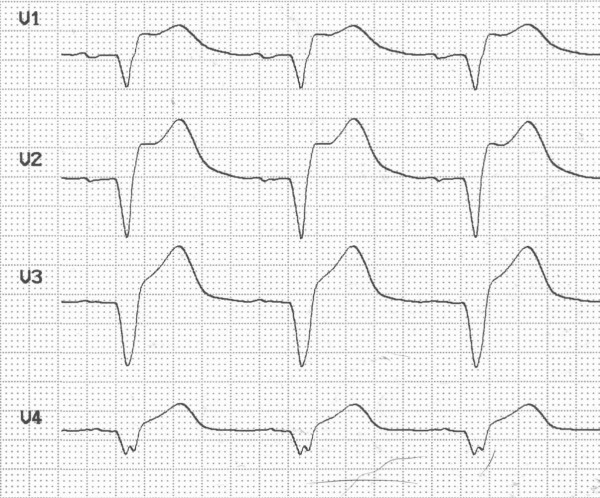
**Electrocardiogram recorded sixteen hours after the accident showing ST-elevations in the anterior leads**.

**Figure 4:**
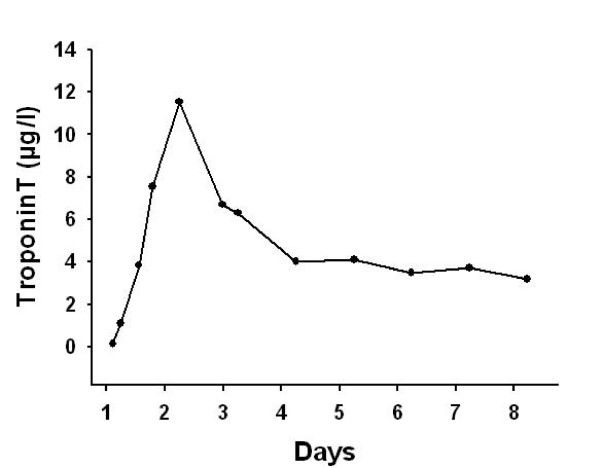
**Serum TnT-levels on admission and daily the first seven days of hospitalisation**.

In traumatic AMI, the diagnosis might be masked by chest pain originating from other thoracic injuries. ECG may be normal [18], but usually demonstrates abnormalities [15,16,19]. Our patient presented with right bundle branch block (Figure 2). In the case of AMI from coronary artery occlusion, ST-elevations, R-loss and Q-wave development are likely to occur [5,8,9]. In our patient, ST-elevations were first recognized sixteen hours after the trauma in the anterior leads (Figure 3). Prior to this our patient developed hypotension (80/50 mmHg) and compromised peripheral circulation. Echocardiography demonstrated marked apical akinesia and slightly dilated left ventricle with ejection fraction (EF) of approximately 30%. There were no signs of valvular injury or hemopericardium. The condition was in our case first perceived as severe cardiac contusion. Echocardiography may show regional motion abnormalities in case of ischemia and AMI [5,9,14,15]. It might also demonstrate hemopericardium and valvular insufficiency [20], if present. Troponin is a sensitive marker of cardiac injury and may be elevated in traumatic coronary artery dissection [8,9]. The pathological increase may develop several hours after admission [13]. In our patient troponin-T was slightly elevated the first hours after admission and reached a maximum of 11.5 μg/L 30 hours after the accident (Figure 4). Both coronary artery occlusion and dissection without occlusion may be demonstrated by a coronary angiogram [3]. If coronary angiography and revascularization is performed early after onset of ischemia, AMI may be avoided [21]. The time lapse from injury to coronary artery occlusion may vary. AMI has been reported to occur immediately and up to five weeks after trauma [5,11,22].

Coronary artery injury may cause AMI, but the natural history of intimal rupture or dissection is not known. Spontaneous healing of the vessel has been described with some degree of residual stenosis [23] and without sequelae [19]. Development of persistent angina pectoris following blunt trauma has been attributed to coronary artery injury in three cases [3,11,24]. Development of coronary artery aneurysm has also been reported [22].

AMI from blunt chest trauma has been managed in several ways. Conservative treatment with inotropic support, if necessary, has resulted in post-infarction sequelae with reduced ejection fraction and cardiac symptoms [25]. Fibrinolytic therapy has been given after mild trauma [17]. Acute percutaneous intervention (PCI) both without [26] and with stent implantation has been performed with successful revascularization and reversal of ST-elevations [21] although restenosis has been described [16]. In our patient PCI was performed and a stent was implanted. As the condition was perceived as cardiac contusion and coronary artery injury was not suspected initially, cardiac catheterization and PCI was performed on the fourth day, after the AMI had taken place. Recovery was uneventful, however, and our patient was fully rehabilitated. Coronary artery bypass grafting has been performed acutely [27] and delayed in combination with coronary aneurysm repair [22] or resection of left ventricular aneurysm and coronary embolectomy [1]. In the multi-traumatized patient off-pump coronary artery bypass (OPCAB) is probably favourable over on-pump surgery [14]. OPCAB is performed without the use of cardiopulmonary bypass resulting in a less coagulopathic procedure. For patients with head injury cardiopulmonary bypass may be a particular risk as cerebral perfusion might be reduced. Avoiding cardiopulmonary bypass might also reduce the risk of organ failure. Moreover, avoiding cardioplegic arrest might be favourable in the case of cardiac contusion since myocardial ischemia also may contribute negatively.

## Conclusion

The possibility of coronary artery injury should be kept in mind after blunt thoracic trauma. This condition probably is underdiagnosed being misinterpreted as cardiac contusion. Modern principles of coronary artery revascularization make myocardial salvage possible, also in the traumatized patient. Following a case of initially overlooked traumatic coronary artery dissection which resulted in AMI we have changed our diagnostic algorithm after blunt chest trauma. ECG is recorded from every patient together with cardiac enzymes. An abnormal ECG and/or abnormal cardiac enzymes warrant further investigation. Both echocardiography and coronary angiography are used when appropriate. The time span from coronary artery occlusion to revascularisation must be short if AMI is to be avoided.

## Competing interests

The authors declare that they have no competing interests.

## Authors' contributions

All authors contributed in the treatment of the patient and in the preparation of the manuscript.

## Consent

The patient has given consent for the case report to be published.
